# Image biomarkers and explainable AI: handcrafted features *versus* deep learned features

**DOI:** 10.1186/s41747-024-00529-y

**Published:** 2024-11-19

**Authors:** Leonardo Rundo, Carmelo Militello

**Affiliations:** 1 Department of Information and Electrical Engineering and Applied Mathematics (DIEM), University of Salerno, Fisciano, Salerno, Italy; 2 High Performance Computing and Networking Institute (ICAR-CNR), Italian National Research Council, Palermo, Italy

**Keywords:** Biomarkers, Diagnostic imaging, Machine learning, Neural networks (computer), Radiomics

## Abstract

**Abstract:**

Feature extraction and selection from medical data are the basis of radiomics and image biomarker discovery for various architectures, including convolutional neural networks (CNNs). We herein describe the typical radiomics steps and the components of a CNN for both deep feature extraction and end-to-end approaches. We discuss the curse of dimensionality, along with dimensionality reduction techniques. Despite the outstanding performance of deep learning (DL) approaches, the use of handcrafted features instead of deep learned features needs to be considered for each specific study. Dataset size is a key factor: large-scale datasets with low sample diversity could lead to overfitting; limited sample sizes can provide unstable models. The dataset must be representative of all the “facets” of the clinical phenomenon/disease investigated. The access to high-performance computational resources from graphics processing units is another key factor, especially for the training phase of deep architectures. The advantages of multi-institutional federated/collaborative learning are described. When large language models are used, high stability is needed to avoid catastrophic forgetting in complex domain-specific tasks. We highlight that non-DL approaches provide model explainability superior to that provided by DL approaches. To implement explainability, the need for explainable AI arises, also through *post hoc* mechanisms.

**Relevance statement:**

This work aims to provide the key concepts for processing the imaging features to extract reliable and robust image biomarkers.

**Key Points:**

- The key concepts for processing the imaging features to extract reliable and robust image biomarkers are provided.
- The main differences between radiomics and representation learning approaches are highlighted.
- The advantages and disadvantages of handcrafted *versus* learned features are given without losing sight of the clinical purpose of artificial intelligence models.

**Graphical Abstract:**

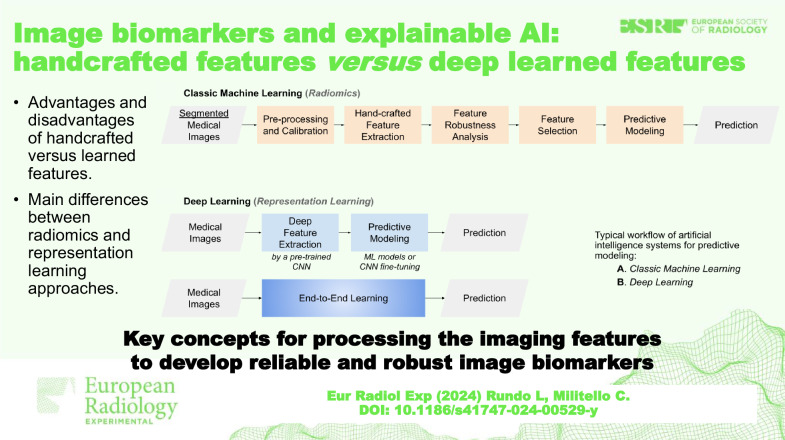

## Background

Feature extraction and selection, along with the most recent approaches in representation learning, are key steps in the image biomarker discovery process, in many areas of clinical research: oncological imaging [1], cardiovascular imaging [2], and neuroimaging [3]. The purpose of this narrative review is to provide an outline of the main types of methods used in the literature for implementing feature selection. Considering that the literature continuously proposes new methods or improvements of existing methods, providing an exhaustive view would have been impossible, as well as beyond the scope of this paper; it is up to the reader, to investigate the different versions/variants belonging to the specific method. Moreover, an *a priori* assessment of the most suitable feature selection method is difficult, and for this reason, often a ‘trial-and-error’ approach is exploited [4, 5].

In precision oncology [6, 7], significant advances concerning the definition/identification of new quantitative biomarkers have been obtained through advanced modeling [8] and multimodal data integration [9]. The integration of information coming from different sources makes it possible to improve performance in machine learning (ML). This aspect is particularly true in healthcare applications where clinical and imaging data can be combined. This novel wealth of information that may now be achieved offers unprecedented potential for implementing precision medicine strategies [10] and optimizing the healthcare workflow [11]. However, this type of biomedical image analysis poses unique challenges that must be handled by specific computational approaches [12]. Artificial Intelligence (AI) is emerging as a transformative force in biomedical imaging analysis and has the potential to provide specific support in decision-making processes, enabling strong cooperation between humans and machines, along with performance assessment [13] and clinical decision-making support [14]. Human and machine perceptions are different and sometimes lead to inconsistent results. The case study reported by Makino et al [15] showed that although it is unclear whether humans and deep neural networks (DNNs) use different features to detect microcalcifications, for soft tissue injuries, DNNs rely on high-frequency components ignored by radiologists.

The extraction and computed analysis of imaging-derived features are mandatory phases for proposing accurate and reliable predictive models to be translated and deployed into clinical environments [16]. In this narrative review, we describe: the fundamentals for processing imaging features to extract reliable and robust image biomarkers; the differences between radiomics based on handcrafted features and representation learning approaches based on learned features; and the advantages and disadvantages of handcrafted *versus* learned features, without losing sight of the clinical purpose of AI models.

## Handcrafted features *versus* learned features

Concerning feature extraction and selection, the main issues to consider are the following:

- All extracted features might be dependent on the data source (clinical or imaging data acquisition centers), thus requiring a harmonization step for dealing with distribution shifts in multicenter studies;
- Handcrafted features are strongly dependent on annotations and may not represent the best choice in complex scenarios;
- Learned features, which are directly extracted from deep learning (DL) architectures, generally maximize performance but are affected by the lack of interpretability in clinical practice, thus requiring explanation methods.

When the problem at hand involves an image classification task, the devised classifier has to take as input some data of interest and provide the output. In the setup phase to define the classifier input, there are two options [17, 18]: (1) using the raw data, as the original pixel/voxel values; (2) extracting features from the image employing well-defined mathematical formulations. Choosing the second approach (extracted features), it is possible to:

- Arbitrarily and manually define a set of features, even though we do not know *a priori* if the selected features are appropriate for the specific classification task (this approach has been used for many years by using classic ML techniques after the feature extraction step);
- Train an ML model to extract and identify useful features for the specific classification task: this approach has become predominant with the advent of DL, which can learn the optimal set of features [19].

More precisely, since the learned features are extracted automatically to solve a specific task, they are extremely effective at it, typically allowing DL models to outperform classic ML models based on handcrafted features manually extracted by the data scientist or AI engineer [20]. This trend is most often observed when comparing ML and DL, but we note that, depending on the scenario and data, classical ML allows for comparable performance to DL architectures.

Regardless of the approach used (ML or DL), the type and distribution variability of input/output data can affect the operation and, consequently, the model performance. ML techniques used in computer-aided medical image analysis usually suffer from the domain shift problem, caused by the different distributions between the source/reference data and the target data. This aspect, which is very evident in multicenter studies, must be considered. To this end, domain adaptation has attracted considerable attention in recent years [21].

While there is the advantage of obtaining features learned automatically by the classifier and being able to develop effective models, there is no control over which features the model will extract from the data and their meaning. Deep features, another way learned features are called because they are intrinsically related to the convolutional layers of convolutional neural network (CNN) architectures [22], are effective for the task under consideration. However, they do not always provide a direct real-world interpretation while the scientific and medical community is devoting its attention to explainable approaches [23].

It is generally difficult to identify the most suitable approach (handcrafted *versus* deep features), and researchers often experimentally compare both alternatives [17].

For the sake of completeness, we highlight that ML algorithms can be categorized into two fundamental types: (1) supervised learning; and (2) unsupervised learning. In supervised learning, there is always a specific measure to be predicted: the target, which represents the dependent variable. All other variables—the ones used to predict what the target will be—are called features and are the independent variables given as input to the model. Depending on the nature of the target variable, you can identify two scenarios of learning that require different learning algorithms within the supervised family: (1) when the target variable is a numeric measure, you need a regression algorithm; (2) when the target variable is a categorical measure, you will need a classification algorithm. In unsupervised learning, the objective is not to make a prediction but to unveil some hidden structure in your data. Unsupervised ML algorithms are capable of exploring your data to find some interesting patterns, a finality that is often called clustering [24].

### Radiomics

Leveraging medical imaging, radiomics is a technique allowing the extraction of features [25] that can be mined to noninvasively assess the *in vivo* phenotype of lesions or even just certain tissue parts (*e.g*., the normal tissue surrounding a tumor) [26–28]. The segmentation of the regions of interest (ROIs) or volumes of interest (VOIs) containing the area/volume from which to extract radiomic features—a process that can be performed through manual or computer-assisted (automatic/semiautomatic) procedures. The predictive models using traditional ML techniques begin with the extraction of large-scale handmade radiomic characteristics: morphometric features (*e.g*., size, shape, diameter measurements) and function quantifying tissue textures (*e.g*., first-order, second-order, higher-order, and transformed domain descriptors). Starting from an input ROI/VOI, the radiomic features can be calculated in two ways: (1) voxel-based extraction (for each feature, a value is computed for each voxel, thus yielding feature maps as output); (2) segment-based extraction (a single, aggregated value per feature is computed for each ROI/VOI). The main classes of handcrafted features are listed in Table 1.

**Table 1:** Classes of handcrafted features along with their types and descriptions

Class of handcrafted features	Types and details
First-order	Histogram-derived features [93]: describe the distribution of voxel intensities within the image ROI
Second-order	Gray-level co-occurrence matrix (GLCM) features: quantify the spatial relationship between pixels, revealing homogeneity, uniformity, linear dependencies, and randomness [94, 95]
Higher-order	Gray-level run-length matrix (GLRLM) features: quantifies gray-level runs, which are defined as the length in number of pixels, of consecutive pixels that have the same gray-level value [67, 96, 97] Neighboring gray-level dependence matrix (NGLDM): quantify pixel properties invariant under rotation; and linear gray-level transformation, useful for texture description and classification [98] Neighborhood gray-tone difference matrix (NGTDM): quantifies the difference between a gray value and the average gray value of its neighbors within a specific distance, able to approach human perception [99] Gray-level size zone matrix (GLSZM): quantifies gray-level variations and homogeneity of image zones Gray-level distance zone matrix (GLDZM): assesses zones of neighboring pixels or voxels with the same gray level and at the same distance from the ROI boundary [100] Gray-level dependence matrix (GLDM): quantification of gray-level dependencies [98]
Transformed domain	Absolute gradient matrix (AGM): texture extraction and analysis Histograms of oriented gradients (HOG): considering the occurrences of gradient orientation in localized portions of an image [101] Local binary patterns (LBP): assign binary numbers to each pixel in an image by thresholding its surrounding pixels [102] Gabor transform [103] Wavelet transform [104]

Although radiomic features are well-known among handcrafted features in the Computer Vision community [25, 29], there are still serious concerns about their stability and robustness [30, 31]. Indeed, radiomic features suffer from a lack of robustness against imaging parameters (*e.g*., spatial resolution) [32] and image extraction settings (*e.g*., quantization levels, resampling) [33–35]. Furthermore, also the software used to extract radiomic characteristics may have an impact on them [36].

The Image Biomarker Standardisation Initiative (IBSI) [29], which provides definitions and nomenclature of radiomics characteristics and defines computation and normalization procedures, tries to resolve/alleviate outstanding challenges in this area. The IBSI also offers implementation recommendations for the various steps of a radiomic workflow, such as intensity discretization, re-segmentation, postacquisition image processing, segmentation, data interpolation, and data conversion in standardized units [37]. Furthermore, for every feature in a model based on binary classifiers, at least ten samples (*i.e*., patients) would be required, according to a well-known rule of thumb [25]. Consequently, adding more features could exacerbate the curse of dimensionality issue by adding redundancy to the features, particularly when there are not enough samples available.

**Fig. 1:**
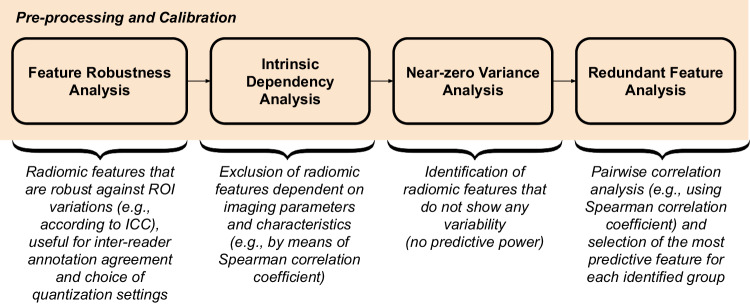
Preprocessing and calibration of radiomic features is a mandatory step to obtain a subset of features that are independent of the region/volume of interest segmentation and the imaging acquisition and reconstruction parameters, which are relevant from the point of view of information content, and nonredundant features. ICC, Intraclass correlation coefficient

To establish robust imaging biomarkers, feature selection procedures specific to the radiomics domain must be developed once features have been calculated and normalized [1]. To achieve this, the selection process should remove: (1) unreliable features (using the intraclass correlation coefficient, for example); (2) features that are not informative based on zero or nearly zero variance; and (3) redundant features (such as those that have a high correlation with one another). Figure 1 outlines the typical preprocessing and calibration steps required by a robust radiomics pipeline as outlined in the following paragraphs.

#### Feature robustness analysis

It is aimed at identifying robust features, for example, considering the effect of variability of the ROIs, *i.e*., the natural situation due to intra- and inter-reader dependence during manual contouring [38]. Moreover, also the optimal quantization setting can be evaluated by extracting the radiomic features considering different quantization levels in terms of either number of bins or bin width. According to the IBSI guidelines, the former (the number of bins) is recommended for calibrated imaging ranges (*e.g*., Hounsfield units in computed tomography, standardized uptake value in positron emission tomography), while the latter (the bin width) is generally used in non-standardized ranges (*e.g*., images obtained through common non-quantitative magnetic resonance imaging sequences). The intraclass correlation coefficient analysis can be used to take into account the extracted features and allows us to determine which are more robust as the perturbations vary and the number of bins varies [39].

#### Intrinsic dependency analysis

Imaging characteristics, and consequently the extracted features, can vary as a function, for example, of magnetic resonance imaging acquisition parameters, such as scanner type, scanner setting, and imaging protocols [40]. For this reason, a correlation analysis (*e.g*., using the Spearman correlation coefficient) allows us to evaluate which features are correlated with, and perhaps dependent on, the imaging acquisition parameters and to select only those features that do not appear to be dependent on them.

#### Near-zero variance analysis

Its goal is to eliminate characteristics that do not convey high information content. This procedure takes into account a cutoff for the percentage of distinct values across all samples as well as a cutoff for the ratio of the most frequent value to the second most common value.

#### Redundant feature analysis

It is aimed at removing highly correlated features to reduce the redundancy among the features. In the case of a correlation value (*e.g*., Spearman correlation coefficient) higher than a threshold (0.90 is a widely used value), the feature with the highest predictive power is selected. A simple skimming approach can be performed by a univariate logistic regression for predicting the lesion characterization, by removing the feature that achieved the lowest area under the receiver operating characteristic. A more sophisticated approach might rely upon dendrograms for identifying groups (*i.e*., clusters) of highly correlated features.

More recently, the dependence on acquisition and reconstruction parameters, such as different reconstruction kernels in computed tomography, has been very recently addressed by using generative adversarial networks (GANs) for image-to-image translation [41, 42]. By doing so, the heterogeneity of the dataset can be mitigated, or even missing data can be generated across sequences or modalities [43, 44]. GAN-based image synthesis has been also combined with diffusion models [45], which represent the latest developments for unsupervised generative approaches in medical imaging [46]. Data harmonization, which allows for the adjustment of variations in imaging methods that generally produce noise in non-AI imaging research, is also crucial in the context of multicentric and multi-institutional studies. With more details, these techniques maintain the information content of images by normalizing the statistical distributions of the same attributes when they are acquired from other systems, such as in the case of ComBat [47], able to combat the batch effect, and its generalizations [48].

Attempts in pictorial interpretation [49], as well as biological and validation, of the radiomic signatures [50], have been carried out. The main characteristic is the intelligibility of radiomic features. Indeed, shallow learning and explainable methods provide insights into the features driving their decisions, allowing clinicians to validate the reasoning behind the recommendation of the system.

Finally, radiomic feature extraction has shown several advantages over deep feature extraction. It is possible to perform an accurate feature extraction also on moderate sample sizes, while deep feature extraction requires a large database to avoid the overfitting issue [51].

### Deep learning models

**Fig. 2:**
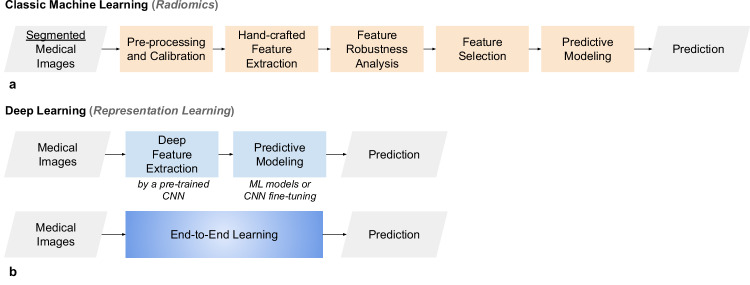
Typical processing pipelines of AI systems for the implementation of predictive models. **a** Classic ML, including the various steps that process handcrafted features. **b** DL exploits representation learning by relying upon deep image feature extraction or end-to-end learning. AI, Artificial intelligence; CNN, Convolutional neural network; DL, Deep learning; ML, Machine learning

With DL models, it is possible to optimize the model’s performance for the given task by automatically extracting image features. DL is a particular sub-field of ML that uses artificial neural networks to interpret raw data directly [52]. To provide the deep architecture with appropriate input, some preprocessing steps must be implemented to condition the data appropriately, and to make the images suitable for deep learning models to process (*e.g*., intensity scaling, resizing, patching, etc). All these processes can impact the performance of the DL model and, for this reason, should be carefully dimensioned and defined case-by-case, depending on the scenario’s data. DNNs make it possible to create end-to-end prediction models by handling every processing step—including feature extraction and learning—that is often required to create a traditional ML model (Fig. 2).

DNNs provide representation learning techniques based on a stack of processing layers with a finite number of nonlinear units (*i.e*., artificial neurons) [53, 54]. Input and output layers are the top and bottom levels of the network, respectively, and the layers in the middle are called hidden layers [55]. DNNs can act as nonlinear function approximators because of their multilayered structure, which allows them to learn many representations of the input data at different levels of abstraction [54]. In contrast, a single neuron acts as a linear classifier.

A DL model can quickly reach millions of trainable parameters to estimate during the training phase, depending on the number of layers and units per layer. As a result, DL models need datasets containing thousands of images, as they are prone to overfitting, especially when working with relatively small training sets [56]. Applications specific to the field of medical imaging encompass both small and big imaging databases, albeit with different consequences. Since DL can predict incredibly complex relationships within large datasets, it has found widespread application in radiation oncology and medical imaging [57].

**Fig. 3:**
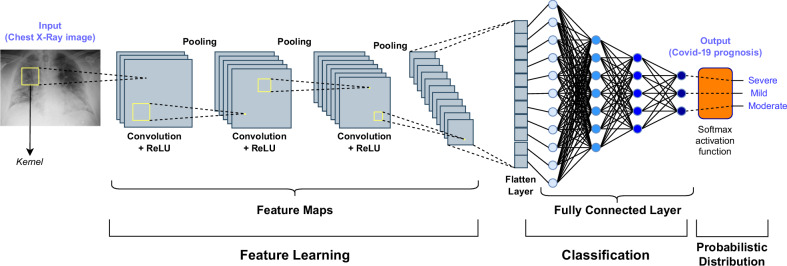
Typical architecture of a CNN for an image classification task. The layers of a CNN learn the features and the final classification is performed *via* a fully connected layer, which processes the feature maps after a flattening operation. In this case, an example of COVID-19 prognosis (*i.e*., three-class classification) based on the analysis of chest x-ray images is depicted [105]. CNN, Convolutional neural network; ReLU, Rectified linear unit

Although ROI/VOI segmentation is not needed for extracting these features (depending on the task at hand), the effectiveness of CNN-based classification might be affected by the different image or patch sizes for including the whole ROI. Importantly, CNNs allow for a hierarchical abstraction of the input data, thus resulting in highly effective signals characterized by a strong spatial/temporal continuity, such as in the case of multidimensional images: CNNs work hierarchically so that the output of one convolution layer is used as input in the next convolution layer. CNN is a type of artificial neural network in which the pattern of connectivity between neurons is inspired by the organization of the human visual cortex, whose individual neurons are arranged in such a way as to respond to the overlapping regions that tessellate the visual field [55]. A typical CNN-based architecture is represented in Fig. 3. According to the architecture shown in Fig. 3, the main components of CNNs are reported in Table 2.

**Table 2:** Main components of a CNN architecture, describing functioning details and processing output

CNN component	Details	Output
Convolutional layers	Kernel size, striding, padding, receptive field. Smaller kernels (3 × 3) with many convolutional layers (~ 1 K) imply fewer parameters than fully connected nets.	Convolutional layers extract features. In general, more than one kernel is applied, thus obtaining a different feature map. Given a square image as input composed of *W*_*in*_ × *W*_*in*_ pixels, *N* different kernels with dimension *K* × *K*, stride *S*, and padding *P*, the output will have dimensions *W*_*out*_ × *W*_*out*_ × *N*, where *W*_*out*_ = [(*W*_*in*_ –*K* + 2 *P*)/*S*] + 1.
Pooling layers	Translation-invariant (preserving important information in local patches). • Max pool (preferable in middle layers) • Average pool (most used in the final layers)	Pooling layers perform a downsampling by dividing the input into regions (*i.e*., pooling windows) and performing an aggregation operation, such as taking the maximum or average value, within each window. This aggregation reduces the size of the feature maps, resulting in a compressed representation of the input data.
Dropout	Destroy/preserve connections with output neurons randomly (with probability *p*_dropout_, which is a hyperparameter used also at test time). • This does not increase the number of parameters and acts as a regularizer • Equivalent to training several smaller networks (in terms of average features from smaller nets) • Training time increases, but it is reduced compared to a multi-expert approach	Dropout performs a regularization for reducing overfitting and improving the generalization of CNNs. Connections are dropped out with a rate *p*_dropout_ at each step during training time.
Activation functions	Sigmoid, softmax, linear, rectified linear unit (ReLU), hyperbolic tangent (Tanh), leaky ReLU	Activation function is used to determine the output of neural network (*e.g*., yes/no, class A/class B, …). The function maps the resulting values in between 0 and 1 or -1 and 1, etc. (depending the used function).
Output layers	Typically fully connected (like in traditional multilayer perceptrons), also called dense layers • Binary classification: one node, sigmoid activation • Multiclass classification: one node per class, softmax activation • Multilabel classification: one node per class, sigmoid activation • Regression: one node, linear activation	Output layers are typically fully connected (FC) layer, called that because each neuron from the previous layer is connected to each neuron of the current layer. FC layers—typically found towards the end of a neural network architecture—are responsible for producing final output predictions.

Tuning the hyperparameters is a nontrivial step after choosing the best network architecture. Structural hyperparameters that can greatly affect model performance include the number of layers/neuronal units, the activation functions, and the receptive field size (the area of the input space that a particular CNN’s feature is interested in). This makes designing the optimal architecture challenging [53].

#### Representation learning

Representation learning was introduced to represent the information efficiently and learn the abstract features from the raw data [58]. These techniques, based on DNNs, are different from handcrafted feature extraction and selection and allow the raw input data to be converted into meaningful intermediate features and outputs [59].

Transfer learning—an ML technique in which a pre-trained model on a task is tuned to a new, related task—can play a crucial role in feature learning, by allowing also for domain adaptation across different institutions [60]. The simplest approach for transfer learning involves “freezing” the first hidden layers (*i.e*., the weights will not be trained, but will keep the values from the previous training procedure) and then applying fine-tuning [61]. Interestingly, shared factors across tasks may also exist, such as in the case of joint segmentation and classification tasks. With many inter-related tasks of interest or many learning tasks in general, each task can be explained by factors that are shared with other tasks, thus allowing for the sharing of statistical strengths across inter-related tasks [58].

CNNs have also been used in conjunction with recurrent neural networks to extract temporal and spatial information from imaging data series. These networks allow the processing of new data (such as longitudinal image series of arbitrary length) while keeping track of previous inputs and outputs since they share node weights throughout time. However, because model complexity is directly correlated with the amount of input data, recurrent neural networks are challenging to train and prone to overfitting.

## Feature selection and dimensionality reduction

In ML, we often incur the so-called problem of the curse of dimensionality, where the number of data samples (*e.g*., patients) is substantially lower than the number of features processed. This could cause problems because it calls for training a large number of parameters on a sparse dataset, which increases the risk of overfitting and suboptimal generalization. However, high dimensionality also leads to extremely long training durations. Therefore, to solve these problems, dimensionality reduction techniques are often used in addition to feature selection. Moreover, while residing in an initial high-dimensionality space, the final space of features has a lower-dimensional structure.

Dimensionality reduction is the transformation from a high-dimensional space (*i.e*., the dataset space) into a low-dimensional space so that the low-dimensional representation retains only meaningful properties of the original dataset. In radiomic-based approaches, after the preprocessing and calibration steps described in Fig. 1, a subsequent selection step is carried out aimed at identifying the most relevant predictive features [62, 63]. In quantitative imaging and radiomics, this approach is preferred to dimensionality reduction since the input features are preserved and are not involved in any transformation process [64]. This aspect is crucial in the development of interpretable models that rely upon the meaning of handcrafted radiomic features.

The high dimensionality of datasets in radiomic investigations is a significant issue, resulting from limited sample numbers and a large number of generic features retrieved from the VOI. To eliminate unnecessary and redundant features, feature selection techniques are applied. Given the abundance of feature selection algorithms available, it is critical to comprehend each one’s effectiveness in the context of radiomics [65].

All of these methods can address the “curse of dimensionality” and decrease overfitting in the model, improving the model’s capacity for generalization. Three classes of feature selection techniques exist:

(i) Filter methods, which evaluate a feature subset’s usefulness using information theory-based metrics or statistical correlation;
(ii) Wrapper methods, which use a search method (such as recursive feature elimination, sequential feature selection, or metaheuristics) to evaluate feature combinations and maximize the predictive model’s performance;
(iii) Embedded methods, which enable feature selection during the model’s training, as in the cases of Elastic net regularization techniques (ElasticNet) and Least Absolute Shrinkage and Selection Operator (LASSO).

Among these, wrapper methods are powerful but computationally demanding [66]. To find the best feature subset, they rely on the evaluation of classification performance. Since exhaustive search methods are computationally intensive and impractical for large-scale datasets, search methods and metaheuristics are typically used to find suboptimal solutions in the search space [67]. Most importantly, repetitive statistical comparisons may create overfitting in the feature subset space as a result of the repeated accuracy estimation utilized in feature subset selection, which would hinder generalization skills [68]. The quality, diversity, and quantity of the data used can directly impact the reliability of the results obtained and can limit the model’s generalization ability. Indeed, medical imaging tasks are typically affected by noise, missing data, and class imbalance since pathological samples represent the minority class compared to healthy samples [69]. Therefore, resampling methods are fundamental for handling missing data during the data curation phase, as well as for dealing with highly unbalanced data during the ML-based modeling phase (*i.e*., data augmentation *via* minority class oversampling) [70].

The output of the selection processes is a set containing relevant, nonredundant, and robust features. The next step of the pipeline is the definition of the predictive model. Depending on the specific clinical topic at hand, multivariable classification or regression techniques can be used to do this [71], usually in supervised learning environments. Interestingly, unsupervised feature selection methods [72] are effective and robust in radiomics applications [73].

The training durations, stability, and similarity of feature selection techniques varied significantly [65]: no single prediction technique was able to consistently outperform the others. According to these findings, less complicated techniques outperform more complicated ones in terms of the area under the receiver operating characteristic curve [74]. They are also more stable. In terms of predictive performance, analysis of variance, LASSO, minimum redundancy, and maximum relevance ensemble seem well-suited for radiomic research, as they outperformed the majority of other feature selection techniques.

### Principal component analysis

To acquire lower-dimensional data while retaining as much of the variation in the data as feasible, the principal component analysis is frequently employed for dimensionality reduction [75]. This is achieved by projecting each data point onto the first principle components only. A direction that optimizes the projected data’s variance can be used to define the first main component. Generally speaking, the direction orthogonal to the first (*i*–1) main components that maximizes the variance of the projected data is the principal (*i*-th) component.

To summarize, principal component analysis learns a linear transformation by projecting the input data onto another space. By limiting dimensionality to some components according to the explained variance of the dataset, dimensionality reduction can be achieved.

### t-distributed stochastic neighbor embedding (t-SNE)

The t-SNE is a nonlinear dimensionality reduction technique designed to effectively embed high-dimensional data for visualization in a two- or three-dimensional low-dimensional environment [76, 77]. To be more specific, every high-dimensional object is represented by t-SNE as a two- or three-dimensional point, with a high probability of representing related objects by nearby points and dissimilar objects by distant points. There are two primary steps in the t-SNE algorithm: (1) creation of a probability distribution between pairs of high-dimensional objects in which the likelihood of similar objects is higher and the probability of different points is lower; (2) establishment of an equivalent probability distribution on the low-dimensional map points and reduction of the Kullback-Leibler divergence between the two distributions concerning the map points’ locations.

The original algorithm bases its similarity metric on the Euclidean distance between objects, although this can be changed as needed.

### Uniform manifold approximation and projection (UMAP)

Similar to t-SNE, the UMAP technique [78] reduces nonlinear dimensionality and can also be applied to generic nonlinear dimension reduction. The three basic assumptions of the UMAP algorithm (concerning data within the Riemannian geometry) are (1) uniform distribution of the data; (2) locally constant Riemannian metric (or approximable); and (iii) locally connected manifold.

### Autoencoders

Autoencoders are an important kind of DL architecture that may be used to reduce the input into a low-dimensional latent space [79, 80]. These networks use progressively smaller hidden layers in the encoder path, regularization, and sparsity constraints to enable learning a lower-dimensional representation of the data, preventing the network from learning the identity transformation, which copies the source data into the destination data without alteration (*i.e*., the trivial solution) [57].

By stacking several nonlinear transformations, each autoencoder layer handles a different transformation. Their design consists of an encoder-decoder, in which the input is mapped to latent space by the encoder and then reconstructed by the decoder. The back-propagation process is used to train them so they can correctly reassemble the input. Autoencoders can be used for dimensionality reduction when the latent space has smaller dimensions than the input. It makes sense that the most significant characteristics of the particular application are encoded by these low-dimensional latent variables, which are discovered during the reconstruction process.

## Conclusions

We summarized and discussed the main aspects of feature extraction and selection with a particular interest in the extraction of reliable and robust biomarkers. We have shown that there is no unifying technique yet. Therefore, despite the outstanding performance of DL methods in many medical image analysis tasks, the use of either handcrafted or learned features needs to be carefully considered for each different study. Radiomics-powered analyses still play a key role in clinically feasible and interpretable applications, allowing for studies that rely on datasets with a limited number of cases.

The size of the dataset is a key aspect: obtaining datasets with too many or too few cases does not represent an optimal situation for model setup. In fact, large-scale datasets (with low sample diversity) could lead to model overfitting; conversely, datasets with limited sample sizes can provide unstable models. Each application scenario must be evaluated in-depth to define the amount of data needed to obtain a well-trained and reliable model. Undoubtedly, the dataset must be representative of all the ‘facets’ of the clinical phenomenon (*i.e*., disease) under investigation.

The access to the computational resources (*i.e*., hardware and software) needed for methods requiring high computational performance hardware—which only graphics processing units can provide—could be a limitation for rapid and broad implementation of the studies (and for the proposed methodologies). This problem is mostly evident in the training phase (once trained, computational demands decrease significantly) and for solutions that rely on deep architectures. However, it must be pointed out that the market now offers very high-performance graphics processing units at affordable prices and programmable with different software, many of them open-source.

Data engineering of large-scale datasets will be fundamental to developing accurate, generalizable DL-powered methods in the near future. A new perspective to avoid data sharing and privacy protection is the federated learning paradigm, also known as collaborative learning, an ML technique allowing an algorithm to be trained through the use of decentralized devices or servers that store data [81, 82]. It enables multi-institutional and reliable studies, along with appropriate data harmonization techniques for information fusion [83]. This aspect has to be taken into account. In fact, it could be very useful in healthcare applications where, because of the sensitive data used, data managers (*e.g*., hospitals) put constraints on data transfer [84]. Moreover, features that are peculiar and well-established for image biomarkers might be effectively supported by large language models (LLMs) [85] in the case of interactive diagnostic tasks powered by AI tools [86, 87].

Indeed, the introduction of LLMs could effectively support diagnostic tasks in quantitative imaging, such as radiology reporting [88], after careful evaluation [89]. LLMs exhibit great performance in language understanding and generation. However, when LLMs are fine-tuned on complex domain-specific tasks, their inference performance on past/historical tasks decreases dramatically [90]. This drawback—called catastrophic forgetting—refers to a phenomenon where an LLM tends to lose previously acquired knowledge as it learns new information. This aspect represents a ‘drift’ for the model and must be addressed to have LLM with stable performance [91, 92].

In conclusion, shallow-learning approaches can provide model explainability that is difficult to achieve with deep architectures. This criticality stems from two different aspects: (1) the lack of interpretability of learned features and (2) the cryptic operation mechanism of deep architectures. So, in DL models, in the face of better performance, we go to a loss of explainability. From this issue, the need for explainable AI arises, which aims to implement explainability (*e.g*., through *post hoc* mechanism) within ML models.

## Abbreviations

AI: Artificial intelligence
CNN: Convolutional neural network
COVID-19: Coronavirus disease 2019
DL: Deep learning
DNN: Deep neural network
IBSI: Image Biomarker Standardization Initiative
LLM: Large language model
ML: Machine learning
ROI: Region of interest
t-SNE: t-distributed stochastic neighbor embedding
UMAP: Uniform manifold approximation and projection
VOI: Volume of interest
